# Supplementary material for: Pituitary Adenylate Cyclase Activating Polypeptide (PACAP) Reduces Oxidative and Mechanical Stress-Evoked Matrix Degradation in Chondrifying Cell Cultures
Source: Int J Mol Sci. 2019 Jan 4;20(1):168. doi: 10.3390/ijms20010168 (PMC6337298; doi:10.3390/ijms20010168)

Pituitary adenylate cyclase activating polypeptide (PACAP) reduces oxidative and mechanical stress-evoked matrix degradation in chondrifying cell cultures

Eszter Szentléleky[a], Vince Szegeczki[a], Edina Karanyicz[a], Tibor Hajdú[a], Andrea Tamás[b], Gábor Tóth[c], Róza Zákány[a], Dóra Reglődi[b], Tamás Juhász[a]*

Supplementary Figures

Apoptotic/Necrotic Cells Detection

100 µL droplets of limb bud mesenchymal cells of different experimental groups were cultured on the surface of 3 cm diameter round coverglass placed into the wells of six-well culture plates. High density cultures were established and after treatment with PACAP, H_2O_2 and MS on day 3 of culturing Apoptotic/Necrotic Cells Detection Kit (PromoCell GmbH, Heidelberg, Germany) was used to detect apoptosis with annexin V labeled with FITC and necrosis with ethidium homodimer III labeled with red fluorescence. Briefly, cells were washed in binding buffers then 5 µl Annexin V and Ethidium homodimer III was added to each well at room temperature for 15 min in dark chamber. Cells were washed and covered with antifade reagent. Photomicrographs were taken using an Olympus DP72 camera on a Nikon Eclipse E800 microscope (Nikon Corporation, Tokyo, Japan).

Supplementary Figure 1. Apoptotic/Necrotic process during PACAP and/or oxidative stress and/or mechanical stress (MS) in high density cultures. Original magnification was 20×. Scale bar: 50 µm. Representative data of 3 independent experiments.

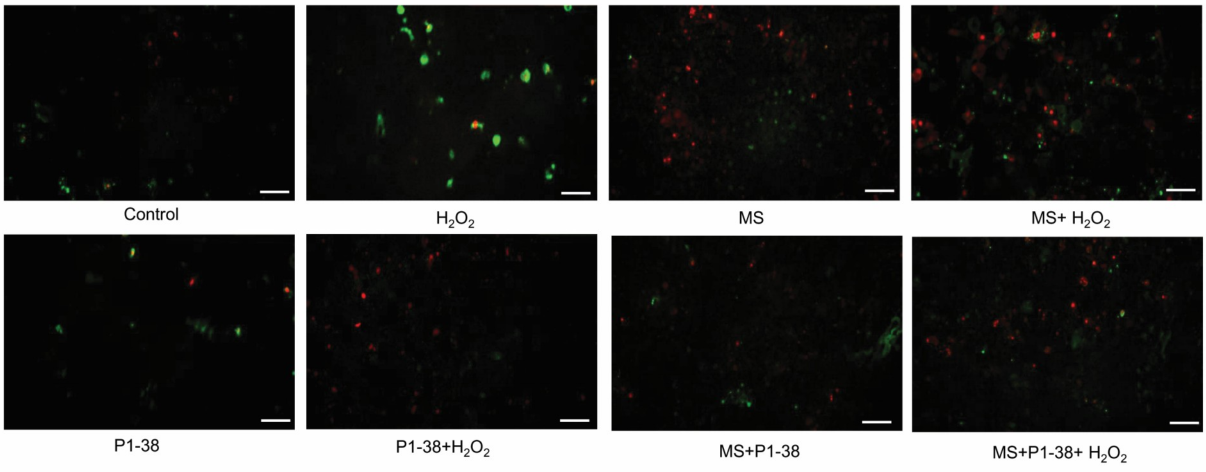

Supplement: Supplementary file 1 [file ijms-20-00168-s001.pdf]
